# Quality of Sleep and Work Productivity among White-Collar Workers during the COVID-19 Pandemic

**DOI:** 10.3390/medicina58070883

**Published:** 2022-07-01

**Authors:** Emilijus Žilinskas, Kristijonas Puteikis, Rūta Mameniškienė

**Affiliations:** 1 Faculty of Medicine, Vilnius University, 03101 Vilnius, Lithuania; emilijus.zilinskas@mf.stud.vu.lt (E.Ž.); kristijonas.puteikis@mf.stud.vu.lt (K.P.); 2 Center for Neurology, Vilnius University, 08661 Vilnius, Lithuania

**Keywords:** absenteeism, anxiety, lockdown, presenteeism, sleep locus of control

## Abstract

*Background and Objectives*: The COVID-19 pandemic has disrupted routine sleep and work patterns in the general population. We conducted an anonymous online survey among white-collar workers from various finance, IT and technology companies in Lithuania to define factors associated with worse sleep quality and diminished productivity during a COVID-19 lockdown. *Materials and Methods*: Employees of selected companies in Lithuania completed an anonymous questionnaire online that included the Pittsburgh Sleep Quality Index (PSQI), The Sleep Locus of Control (SLOC), the Generalized Anxiety Disorder Scale-7 (GAD-7), and the World Health Organization’s Health and Work Performance Questionnaire (WHO-HPQ). Respondents also provided information about their sleep hygiene, physical activity and alcohol use. *Results*: Data of 114 respondents (56, 49.1% male) were used for analysis. Among them, 49 (43.0%) suffered from poor sleep and 29 (25.4%) had clinically relevant levels of anxiety. However, there were only negligible levels of absenteeism in the sample (a median of zero hours of work lost over the past month). In a stepwise linear regression model (F(5,108) = 11.457, *p* < 0.001, R^2^_adj_ = 0.316), high levels of anxiety, daily hours spent using the screen, use of electronic devices in the bedroom, smoking in the evening, and COVID-19-related changes in appetite were associated with worse sleep quality. Absenteeism was associated with physical activity of moderate intensity and decreased self-reported productivity during the pandemic (F(2,111) = 7.570, *p* = 0.001, R^2^_adj_ = 0.104). However, there was no strong relationship between sleep-related variables (i.e., sleep hygiene, sleep locus of control, quality of sleep) or levels of anxiety and measures of work productivity. *Conclusions*: Our findings suggest that while bad sleep hygiene, anxiety, and changes in appetite are associated with worse sleep quality among white-collar workers during the pandemic, work productivity may remain high irrespective of disrupted sleep.

## 1. Introduction

Infection spread-associated lockdowns during the COVID-19 pandemic led to remarkable everyday changes in affected populations. A lack of physical activity [1,2], circadian misalignment [3,4], increase in the usage of digital media [5], decreased exposure to daylight, and changes in eating habits [6] may have resulted in a significant deterioration of sleep quality during COVID-19-related quarantines [7,8,9,10,11,12]. Mental distress is another known determinant of sleep quality and it has also significantly increased during the pandemic [13,14,15]. Given the bidirectional relationship between sleep and anxiety, the latter could have a major role in the deterioration of sleep quality during this period [16,17]. A deterioration in sleep quality may also depend on an individual’s attitude towards his/her ability to control personal sleep patterns. For instance, an “internal locus of control”—a person’s perception that outcome on a certain aspect of their health results from their own behaviour rather than external forces or chance—is supposed to lead to better psychological outcomes, particularly during the pandemic [18,19]. However, the relationship between sleep locus of control and sleep quality during the pandemic has been less investigated. Apart from individual attitudes, various external factors such as social isolation, professional hardships, and changes in routine tasks, are important for sleep quality as well [20,21]. Worse sleep may in turn create a feedback loop and result in worse real-world outcomes for the individual. For instance, reduced work productivity may be a possible burden for societies subject to strict lockdown measures [22]. Sleep disturbances may increase the likelihood of negative work outcomes such as absenteeism or occupational accidents [23,24,25], whereas adequate sleep is associated with higher productivity [26,27,28]. Studies evaluating the impact of the COVID-19 pandemic and working from home on employees’ work productivity reveal a complex interaction between individual, organizational, and societal factors in determining the work engagement and employee well-being while working from home [29,30,31,32]. However, there are fewer reports on whether worse sleep quality is directly associated with productivity loss during the pandemic.

We conducted an online survey among white-collar workers to (1) investigate the determinants of sleep quality during the COVID-19 pandemic among employees of finance, IT and technology sectors and (2) evaluate the relationship between sleep-related variables as well as sleep quality with measures of work productivity.

## 2. Materials and Methods

### 2.1. Study Setting

We contacted the administrative staff of different Lithuanian companies from finance, technology, and IT sectors (*n* = 105) via email, requesting them to share an online survey form (created in Google Forms) with their co-workers. Companies were selected from a local internet website (https://rekvizitai.vz.lt/en/ accessed on 28 January 2021) that provides a search engine for businesses registered in Lithuania. A search was conducted by applying an upper limit on the number of employees (*n* < 750), including filters to show companies from finance, technology, and IT sectors, and sorting the results by relevance. The first result pages were then used to select the first 100 companies to approach by messaging. To ensure faster response, only companies having active social media websites (e.g., Facebook) were contacted through their respective platforms. The recruitment process lasted from 1 February to 9 April 2021, during a strict COVID-19-related national lockdown in Lithuania (it was initiated on 7 November 2020). All questions were compulsory for submitting the form. Answers were screened on a case-by-case basis to identify and exclude any inconsistent submissions. The response rate was calculated by dividing the number of respondents by the total number of staff working in all companies who shared the questionnaire with their employees.

### 2.2. The Questionnaire

The questionnaire consisted of several parts:

- General questions regarding the age, sex, height, and weight of the participants, their use of alcohol or tobacco, and incidence of COVID-19 infections or COVID-19-related self-isolation or hospitalization.
- Sleep hygiene. Respondents reported the frequency (on a 5-point Likert scale) of using media in their sleeping room or before bedtime, taking a nap during daytime, smoking or drinking caffeinated beverages or alcohol in the evening.
- The Sleep Locus of Control (SLOC) Questionnaire, which included eight questions measuring the degree to which an individual attributes his or her experiences of sleep to either chance or internal causes [33]. A higher score indicates a more individual-dependent attitude towards sleep.
- The Pittsburgh Sleep Quality Index (PSQI) Questionnaire [34]. This is a 19-item tool which measures sleep quality, sleep latency, sleep duration, habitual sleep efficiency, sleep disturbances, use of sleep medications, and daytime dysfunction. Higher scores indicate worse sleep quality.
- To assess respondents‘ work performance, we used the absenteeism and presenteeism questions of the World Health Organization’s (WHO, Geneva, Switzerland) Health and Work Performance Questionnaire (HPQ) [35]. Absenteeism refers to the habitual non-presence of an employee at their job [36]. In other words, absenteeism may be defined as sickness absence. Within the WHO-HPQ, absenteeism is defined as working hours lost due to sickness absence (a high score indicates a higher number of hours lost, whereas a negative score indicates that the respondent worked more than expected). The measure of relative absenteeism is expressed as a percentage of expected work hours and ranges between a negative number (works more than expected) and 1.0 (always absent). Absenteeism was calculated using 4-week estimates. Presenteeism is a measure of actual work performance in relation to possible performance (a higher score indicates a lower amount of lost performance). Absolute presenteeism varies between 0 (total lack of performance during time on the job) and 100 (ideal performance). Relative presenteeism is a ratio of actual performance to the performance of most workers at the same job. The distribution of relative presenteeism was restricted to the range of 0.25 to 2, where 0.25 is the worst relative performance (25% or less of other workers’ performance) and 2.0 is the best performance (200% or more of other workers’ performance). Large discrepancies were indicative of superficial responding; in such cases, more detailed examination of case-by-case responses was used to make rational decisions about case deletion.
- The Generalized Anxiety Disorder Scale-7 (GAD-7) [37]. Seven items assess the frequency of anxiety symptoms over the past two weeks on a 4-point Likert scale and ≥10 is used as a cut-off for identifying clinically relevant cases of GAD.
- Respondents’ weekly physical activity levels were evaluated based on WHO’s guidelines on physical activity and sedentary behaviour in which at least 150 min of moderate-intensity or at least 75 min of vigorous-intensity physical activity is recommended for adults [38].
- Changes in sleep, appetite, exercise, work productivity and alcohol use during the COVID-19 pandemic were evaluated by using ad hoc questions based on a 5-point Likert scale.

Pre-translated Lithuanian versions of the PSQI (Cronbach’s α = 0.720) and GAD-7 (Cronbach’s α = 0.932) were used, and the SLOC (Cronbach’s α = 0.630) and WHO-HPQ were directly translated by the authors (E.Ž., K.P.) as no identified Lithuanian versions were found.

### 2.3. Statistical Analysis

Statistical analysis was conducted in Microsoft Excel v16 and IBM SPSS v26. Data normality was assessed using tests of Kolmogorov–Smirnov and Shapiro–Wilk as well as an evaluation of Q-Q plots. For ordinal or non-normally distributed data, Spearman’s correlation, Mann–Whitney U or Kruskal–Wallis tests were used. Given the exploratory nature of the survey, there was no adjustment for multiple testing and the threshold for statistical significance was set at *p* < 0.05. Variables related to physical health, sleep hygiene, or COVID-19 that correlated with the PSIQ or the estimate of 4-week relative absenteeism were included in their respective stepwise linear regression models. Afterwards, variables that were statistically significant in the aforementioned stepwise linear regression models were all incorporated in a path analysis in Amos Graphics, which allows visualization of correlation as well as regression coefficients from respective models. We sought a sample size of at least 103 to include up to 7 variables in a linear multiple regression model with α = 0.05, 1 − β = 0.80, and f^2^ = 0.15.

## 3. Results

We contacted 105 companies and the administrative staff of 35 (33.3%) of them agreed to share the questionnaire with their employees. The total number of employees in the 35 companies was 3084. As 131 participants completed the survey form, the overall response rate was 4.2%. Of the 131 individuals who completed the survey, 114 (87.0%) responses were included in further analyses. The general characteristics of these respondents are presented in Table 1. There were 20 (17.5%) smokers who have been smoking an average of 10.9 (±5.7) cigarettes per day for 9.0 (±6.6) years. Respondents drank a median of 5 (0–50) units of alcohol per week and spent 12 (0–17) hours per day interacting with electronic devices that have a screen.

**Figure 1:**
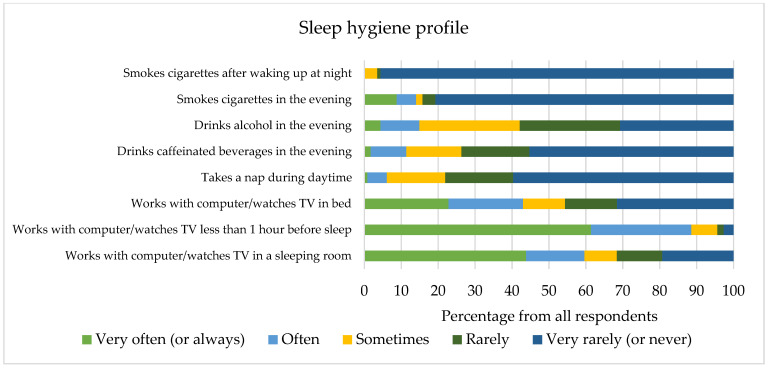
Questions evaluating the sleep hygiene of respondents in our study.

According to the PSQI questionnaire data, 43.0% of respondents suffered from poor sleep (PSIQ > 5). Respondents slept a median of 7 (from 4.5 to 9.5) hours per day during the previous month. General sleep quality did not depend on sex (Z = −0.371, *p* = 0.710) or way of living (H = 2.719, *p* = 0.606). The response rates to sleep hygiene-related questions are presented in Figure 1. The use of media devices in the bedroom and before sleep correlated with higher PSQI scores (r_s_ = 0.196, *p* = 0.037 and r_s_ = 0.188, *p* = 0.045, respectively). Hours spent using devices each day were also associated with higher scores on the sleep questionnaire (r_s_ = 0.197, *p* = 0.036). A relationship was present between PSQI scores and smoking in the evening (r_s_ = 0.211, *p* = 0.024), or during periods of awakening (r_s_ = 0.217, *p* = 0.020). High- but not medium-intensity training was linked to lower PSQI scores (r_s_ = −0.295, *p* = 0.001 and r_s_ = −0.169, *p* = 0.072, accordingly). Symptoms of anxiety were related to worse sleep quality (r_s_ = 0.393, *p* < 0.001). There was no association between measures of either presenteeism, absenteeism, or sleep locus of control, and general sleep quality (*p* > 0.05).

The results of the SLOC questionnaire did not correlate significantly with aspects of respondents’ sleep hygiene habits (*p* > 0.05). A lower SLOC score was associated with worse sleep efficiency (a subscale of the PSQI) (r_s_ = −0.280, *p* = 0.003), but not with general sleep quality, as measured by the total PSQI score (r_s_ = −0.098, *p* = 0.301).

**Figure 2:**
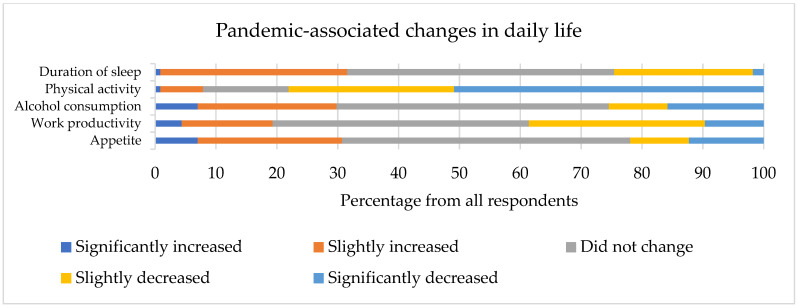
Changes in respondents’ daily lives during the COVID-19 pandemic.

Seven (6.1%) respondents were confirmed to have COVID-19. None of them were hospitalized; however, four (57.1%) experienced long-term symptoms of COVID-19. Their sleep quality and anxiety levels were comparable to those who remained non-infected (Z = 0.989, *p* = 0.369; Z = −0.456, *p* = 0.648, respectively). There were 12 (10.5%) individuals who had to self-isolate during the previous month. The median number of days when respondents worked remotely during the previous month was 12 (0–31). Answers to questions related to daily life changes due to the COVID-19 pandemic are presented in Figure 2. A reported increase in appetite and a decrease in physical activity and sleep duration during the COVID-19 pandemic were associated with higher PSQI scores (r_s_ = 0.242, *p* = 0.009, r_s_ = −0.284, *p* = 0.002, and r_s_ = −0.208, *p* = 0.026, respectively). COVID-19-related variables were not associated with measures of work productivity, apart from a relationship between a self-perceived decrease in productivity during the pandemic and measures of absenteeism (4-week absolute absenteeism: r_s_ = −0.290, *p* = 0.002; 4-week relative absenteeism: r_s_ = −0.298, *p* = 0.001).

According to the GAD-7 questionnaire data, 25.4% of respondents experienced moderate to severe levels of anxiety (≥10 points). A higher consumption of alcohol and reduced physical activity during the pandemic correlated with symptoms of anxiety (r_s_ = 0.187, *p* = 0.046; r_s_ = −0.186, *p* = 0.048, respectively).

Questions from the WHO-HPQ questionnaire revealed that respondents’ work performance during the previous 4 weeks was, on average, equal to 80% of their best possible performance. The evaluation of absenteeism indicated that respondents did not lose any working hours during the previous 4 weeks (median = 0, range from −90 to 125). Forty-seven (41.2%) respondents claimed that they worked more than expected. The measure of presenteeism correlated with daytime dysfunction due to sleepiness (absolute presenteeism: r_s_ = −0.207, *p* = 0.027) and sleep latency (absolute presenteeism: r_s_ = −0.188, *p* = 0.045). Absenteeism was related to daily screen use (4-week absolute absenteeism: r_s_ = −0.233, *p* = 0.013; 4-week relative absenteeism: r_s_ = −0.227, *p* = 0.015) and medium-intensity physical activity (4-week absolute absenteeism: r_s_ = 0.188, *p* = 0.045; 4-week relative absenteeism: r_s_ = 0.182, *p* = 0.052).

**Figure 3:**
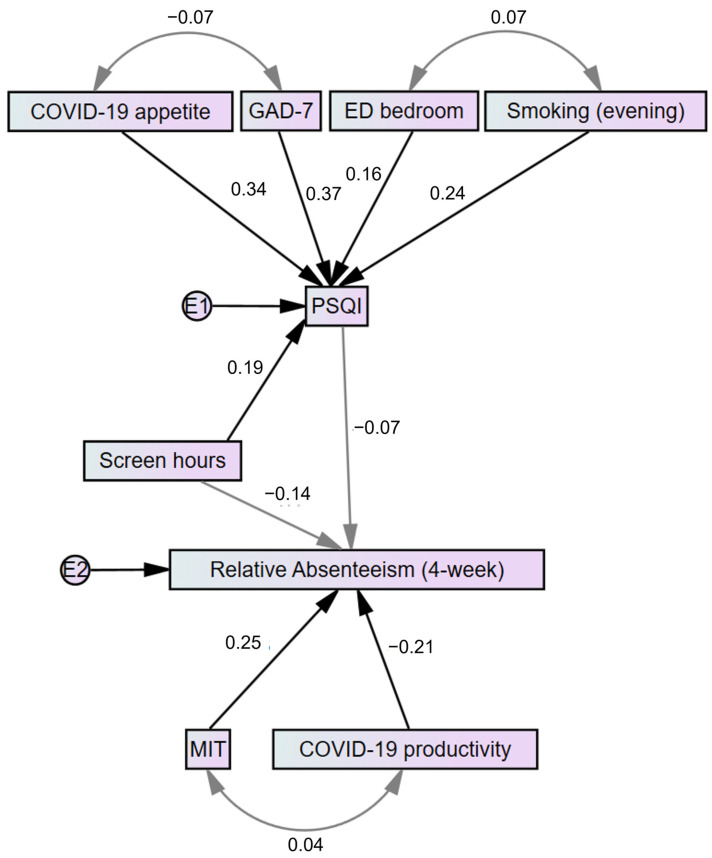
A path analysis representing the main findings regarding sleep quality and absenteeism. Gray-coloured lines indicate relationships that are non-statistically significant. Numbers indicate either beta-coefficients in a respective regression model or correlation coefficients (single and two-headed arrows, accordingly). E—error; ED bedroom—the use of electronic devices in the bedroom; GAD-7—Generalized Anxiety Disorder Scale-7; MIT—medium-intensity training; PSQI—Pittsburgh Sleep Quality Index.

A stepwise linear regression model for PSQI was created by including variables that were previously found to correlate with the PSQI score (F(5108) = 11.457, *p* < 0.001, R^2^_adj_ = 0.316). A respective model with 4-week relative absenteeism as the dependent variable (F(2111) = 7.570, *p* = 0.001, R^2^_adj_ = 0.104) included self-reported productivity during the COVID-19 pandemic, the number of screen hours per day, and the time spent in medium-intensity training (the latter variable was included as its correlation with the measure of absenteeism approached statistical significance). The number of daily screen hours was excluded from the model during stepwise selection (*p* = 0.09). A summarized model of the regression models is presented as a path analysis in Figure 3.

## 4. Discussion

We report the results of an anonymous survey investigating the association between sleep and work productivity among white-collar workers in Lithuania during a COVID-19 lockdown. Currently, there are little data regarding patterns of sleep and work productivity during the COVID-19 pandemic in this population. Recent reports suggest that working remotely may be associated with worse productivity, which itself is related to mental health and sleep problems [39,40,41]. However, our data indicate that sleep quality and work productivity were not directly related to each other and were influenced by different determinants. To the best of our knowledge, no previous studies which used standardized PSQI, SLOC, and WHO-HPQ questionnaires to investigate the determinants of sleep quality and work productivity during the COVID-19 pandemic.

Our results suggest that sleep quality among respondents was suboptimal. Worse sleep quality was associated with bad sleep hygiene (the most prevalent issue was the use of electronic devices before sleep or in the bedroom), anxiety, and appetite changes during the pandemic, but not sleep locus of control. The latter finding partially contradicts evidence that suggests attitudes and beliefs regarding sleep are better predictors of sleep outcomes than sleep knowledge [42]. For example, it may be questionable whether sleep hygiene education programmes actually lead to better sleep [43]. Definitive conclusions are difficult to draw from such reports because of study heterogeneity, and the predominance of studies of childhood or adolescence rather than adulthood [44]. In contrast, attitudes about sleep are seen to be significantly associated with the results of behavioural interventions [42,45,46,47,48,49]. However, neither sleep hygiene habits, nor overall sleep quality were associated with SLOC in our study. This finding suggests that SLOC is a construct that may qualitatively differ from attitudes measured by other instruments, such as the Dysfunctional Beliefs and Attitudes about Sleep questionnaire [50]. Further, as SLOC represents attitudes towards sleep rather than current behaviour, this may underlie our finding that reported factual sleep hygiene habits (e.g., screen use before sleep) rather than SLOC were statistically significant determinants of sleep quality. While SLOC can affect sleep by mediating the impact of cognitive-behavioural therapy on the treatment of insomnia, more research is needed to prove the usefulness of the SLOC scale in relation to sleep quality [51].

Our findings are consistent with the widespread notion that the quality of sleep highly depends on sleep hygiene [52,53,54,55,56,57]. Selected behaviours, such as the use of electronic devices around bedtime, or smoking in the evening, were associated with worse PSQI scores. Therefore, a person may potentially improve his/her sleep during a lockdown by minimizing the use of electronic devices in the evening and abstaining from smoking. Interestingly, increased appetite during the COVID-19 pandemic was the single COVID-19-related factor that contributed to explaining the variance in sleep quality. It may be considered that an alteration in usual meal patterns (e.g., easy snacking at home) subsequently led to disrupted sleep–wake patterns [56]. Therefore, changes in daily eating habits should be recognised as an important factor that may worsen the quality of sleep in the context of a pandemic.

The rate of clinically relevant levels of anxiety (25.4%) was comparable to reports from Europe [58] and, according to our findings, could potentially be reduced by preventing sedimentary routines and excessive consumption of alcohol during the pandemic. Further, the close relationship between anxiety and quality of sleep measured in our study was in concordance with previous research that highlighted high rates of comorbidity between anxiety disorders (for example, generalized anxiety disorder) and sleep illnesses (such as insomnia) [59,60,61]. However, the coexistence of high levels of anxiety and sleep problems is not yet fully explained. It is reasonable to consider that anxiety treatment may be effective in improving one‘s sleep; however, current evidence suggests that treatment implications in a separate manner may more likely lead to a full therapeutic effect [62]. In addition, one systematic review indicated anxiety and sleep to be bidirectionally related [63]. Considering increased levels of anxiety during the current epidemiological crisis, future studies should investigate a possible causal/consequential relationship between anxiety and sleep in more detail.

Our study reveals that, in our sample, the loss of productivity because of absenteeism during the last 4 weeks was minimal if not negligible. Two in every five respondents worked even more than expected. However, the measure of presenteeism revealed that respondents did not work at their maximum capabilities. Such results could indicate that respondents may work additional hours to compensate for the self-perceived submaximal work performance. This trend can become burdensome because of lost time for resting or sleeping. However, our study suggested that presenteeism is associated with only some components of the quality of sleep and none of the other health- or COVID-19-related variables. Respectively, selected items in the survey explained only a tenth of the variance of 4-week relative absenteeism. As only self-perceived work productivity and the physical activity of moderate intensity were significant variables in the model, our study could not substantiate the hypothesis that worse sleep quality was associated with reduced work performance. This finding may be partly confounded by methodological aspects (e.g., work productivity was self-reported rather than measured objectively) and specifics of the respondent group (e.g., relatively young workers may manage to exchange optimal sleep habits for more productive working hours) [64,65]. Further, the formula of calculating absenteeism involves working hours lost because of health problems as well as other reasons. Thus, the association of exercising with higher absenteeism may be explained by respondents using working hours for physical activity while preserving overall work performance (hence, no association between physical activity and presenteeism was identified) [66]. To the best of our knowledge, our study is the first to utilize the WHO-HPQ measures to evaluate the link between sleep and work productivity. Other studies have used instruments such as the Work Limitations Questionnaire [24] or the Work Productivity and Activity Impairment questionnaire [25,26,28]. Thus, larger studies using the WHO-HPQ could better define the relationship between work productivity, sleep, and other health-associated factors.

Our investigation should be interpreted in the context of its limitations. One of them is a small sample size, which can be explained by workers’ hesitation to dedicate their time to completing the survey. This resulted in lower statistical power and indicates a large non-respondent bias (i.e., workers who were disinterested in sleep-related problems had no real incentive to participate in the survey). A relatively small sample size may have also prevented us from proving the association between general sleep quality and work productivity that was expected at the start of study. Further, we chose the SLOC questionnaire because of its brevity and did not include any other scale estimating sleep-related beliefs (e.g., the DBAS-16). The latter could have provided additional evidence regarding the impact of one’s attitudes towards sleep and sleep quality. The anonymous and online design of our study resulted in isolated cases of superficial responding, which could have been prevented by explaining the questions in person. The case-by-case exclusion of such data further decreased the sample size and introduced additional selection bias. Finally, respondents did not report whether they work from home full-time or have in-office hours. While we expect online work to be predominant in our sample because of lockdown measures, selected individuals who spent time working in an office may have influenced our analysis.

## 5. Conclusions

Our study spotlights the challenges young white-collar employees faced during the COVID-19 pandemic. The results provide evidence that poor sleep hygiene habits, high levels of anxiety, and everyday changes in eating patterns were associated with a worse quality of sleep during the COVID-19 pandemic. Except for the level of moderate-intensity physical activity, there was no clear association between other health- or pandemic-associated variables and loss of productivity. Additional research is needed to better define the relationship between sleep habits, sleep quality, physical activity, and work productivity during health crises resulting in strict lockdowns.

## Figures and Tables

**Table 1:** Demographic variables and results from the PSQI, SLOC, GAD-7, and WHO HPQ questionnaires.

Variables	Categories	Result
Number of respondents		114
Age (median, range)		29 (21–46)
Sex (*n*, %)	Male	56 (49.1)
	Female	58 (50.9)
Way of living (*n*, %)	Alone	26 (22.8)
	With parents	15 (13.2)
	With children w/o partner	1 (0.9)
	With partner w/o children	50 (43.9)
	With partner and children	22 (19.3)
Physical activity	Moderate-intensity physical activity (median of minutes per week, range)	120 (0–1320)
	High-intensity physical activity (median of minutes per week, range)	0 (0–360)
	Sufficient physical activity (*n*, %)	57 (50)
Quality of sleep	PSQI score (median, range)	5 (1–14)
Sleep locus of control (SLOC) (median, range)		33 (21–46)
GAD-7 score (median, range)		6 (0–21)
Level of anxiety (*n*, %)	Minimal	45 (39.5)
	Mild	40 (35.1)
	Moderate	17 (14.9)
	Severe	12 (10.5)
Absolute absenteeism (median, range)		0 (−90–125)
Relative absenteeism (median, range)		0 (−0.56–0.78)
Absolute presenteeism (median, range)		80 (30–100)
Relative presenteeism (median, range)		1 (0.5–2)

## Data Availability

Raw data are available from the authors upon reasonable request.
